# Muju Virus, Harbored by *Myodes regulus* in Korea, Might Represent a Genetic Variant of Puumala Virus, the Prototype Arvicolid Rodent-Borne Hantavirus

**DOI:** 10.3390/v6041701

**Published:** 2014-04-14

**Authors:** Jin Goo Lee, Se Hun Gu, Luck Ju Baek, Ok Sarah Shin, Kwang Sook Park, Heung-Chul Kim, Terry A. Klein, Richard Yanagihara, Jin-Won Song

**Affiliations:** 1 Department of Microbiology, College of Medicine, and the Institute for Viral Diseases, Korea University, Seoul 136-705, Korea; E-Mails: jingoo84@gmail.com (J.G.L.); gsehun@korea.ac.kr (S.H.G.); baekmicr@korea.ac.kr (L.J.B.); parkbcmb@korea.ac.kr (K.S.P.); 2 Department of Biomedical Science, College of Medicine, Korea University, Seoul 136-705, Korea; E-Mail: sarahshin79@gmail.com; 3 5th Medical Detachment, 168th Multifunctional Medical Battalion, 65th Medical Brigade, Unit 15247, APO AP 96205-5247, USA; E-Mail: hungchol.kim@us.army.mil; 4 Public Health Command Region-Pacific, 65th Medical Brigade, Unit 15281, APO AP 96205-5281, USA; E-Mail: terry.a.klein2.civ@mail.mil; 5 Pacific Center for Emerging Infectious Diseases Research, John A. Burns School of Medicine, University of Hawaii at Manoa, Honolulu, HI 96813, USA; E-Mail: ryanagih@hawaii.edu

**Keywords:** hantavirus, *Myodes regulus*, Muju virus, phylogeny

## Abstract

The genome of Muju virus (MUJV), identified originally in the royal vole (*Myodes regulus*) in Korea, was fully sequenced to ascertain its genetic and phylogenetic relationship with Puumala virus (PUUV), harbored by the bank vole (*My. glareolus*), and a PUUV-like virus, named Hokkaido virus (HOKV), in the grey red-backed vole (*My. rufocanus*) in Japan. Whole genome sequence analysis of the 6544-nucleotide large (L), 3652-nucleotide medium (M) and 1831-nucleotide small (S) segments of MUJV, as well as the amino acid sequences of their gene products, indicated that MUJV strains from different capture sites might represent genetic variants of PUUV, the prototype arvicolid rodent-borne hantavirus in Europe. Distinct geographic-specific clustering of MUJV was found in different provinces in Korea, and phylogenetic analyses revealed that MUJV and HOKV share a common ancestry with PUUV. A better understanding of the taxonomic classification and pathogenic potential of MUJV must await its isolation in cell culture.

## 1. Introduction

Currently, 23 hantaviruses (genus *Hantavirus*, family *Bunyaviridae*), found in reservoir murid and cricetid rodent species in Eurasia, Africa and the Americas, have been recognized as distinct species by the International Committee on Taxonomy of Viruses (ICTV) [1]. Yet, if one considers the myriad hantavirus sequences deposited in GenBank, including those of previously unrecognized hantaviruses harbored by shrews, moles and insectivorous bats [2], then innovative and collaborative solutions are clearly needed to facilitate timely and accurate classification of newfound and unassigned hantaviruses [3]. Admittedly, several of the unclassified hantaviruses are not distinct species but instead represent genetic variants or genotypes of well-recognized hantavirus species. For example, multiple strains or isolates of Hantaan virus (HTNV), Seoul virus (SEOV), Dobrava virus (DOBV) and Puumala virus (PUUV), some of which have been associated with hemorrhagic fever with renal syndrome (HFRS), are known. Similarly, many examples of Sin Nombre virus (SNV) and Andes virus (ANDV), causing hantavirus cardiopulmonary syndrome (HCPS), have been detected in sigmodontine and neotomine rodent species. On the other hand, many of the recently identified soricomorph- and chiropteran-borne hantaviruses probably represent new species and remain unassigned largely because they have not been isolated in cell culture and exist only as partial or full-length sequences, making their taxonomic classification, using the current ICTV criteria more difficult.

Viral sequences related to PUUV, the prototype arvicolid rodent-borne hantavirus harbored by the bank vole (*Myodes glareolus*), which causes HFRS in Europe, including Scandinavia, where the disease is commonly referred to as nephropathia epidemica (NE) [4,5], have been found in Asian voles. For example, Hokkaido virus (HOKV) has been reported in the grey red-backed vole (*My. rufocanus*) in Japan [6] and Russia [7]. Based on the phylogenetic relationship between *My. glareolus* and other arvicolid rodent species, we previously targeted the royal vole (*My. regulus*) in Korea as a likely reservoir host and detected a hantavirus, designated Muju virus (MUJV) [8]. Amino-acid sequence analysis suggested that MUJV might be distinct from PUUV. Unfortunately, no L-segment sequences were available. Thus, the aim of this study was to obtain the whole genome sequence of MUJV to ascertain its genetic and phylogenetic relationship with HOKV and PUUV.

## 2. Results

**Figure 1:**
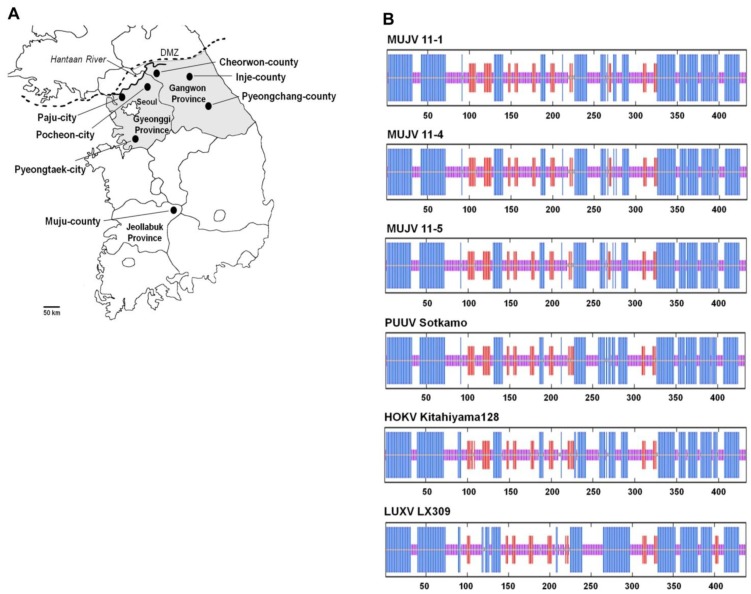
(**A**) Map of Korea, showing sites where royal voles were captured during 2008–2011. Also shown is the location of Muju county in Jeollabuk province, where the original MUJV-infected royal voles were trapped; (**B**) Schematic comparison of consensus secondary structures of the full-length nucleocapsid protein (Np) of MUJV, PUUV, HOKV and LUXV, as predicted using several methods available at the NPS@ structure server [10]. Alpha helices are represented by blue bars, beta-strand by red bars, and random coils and unclassified structures by magenta and gray bars, respectively.

Of 101 royal voles, taxonomically verified by mitochondrial DNA (mtDNA) analysis, captured in six sites (Inje, Pyeongchang and Cheorwon counties in Gangwon province, and Pocheon, Paju and Pyeongtaek cities in Gyeonggi province) during 2008–2011 (Figure 1A), four voles from Inje (11-1, 11-16, 11-19 and 11-21) and two voles from Pyeongchang (11-4 and 11-5) had IgG antibodies against PUUV. The full-length L-, M- and S-genomic segments of MUJV were sequenced for MUJV 11-1, 11-4 and 11-5, and partial sequences were obtained for MUJV 11-16, 11-19 and 11-21 (Table 1). The 5'- and 3'-terminal sequences of the MUJV L, M and S segments, which form panhandle structures based on complementarity, were confirmed by RACE PCR as 5'-UAGUAGUAGACUCC-3' and 3'-AUCAUCAUCUGAGG-5', respectively. A mismatch at position 9 (5'-UAGUAGUAUGC-3') with a noncanonical U-G pairing at position 10, reported previously [9], was not found in any of the genomic segments of the MUJV strains, or in the S segments of PUUV Sotkamo, PUUV CG1820, PUUV Kazan, PUUV Fusong_Cr247, PUUV Fusong_mf682, HOKV Kami-iso 8Cr_95, HOKV Tobetsu 60Cr_93, and several other hantaviruses, as evidenced by sequences available in GenBank.

**Table 1:** Nucleotide and amino acid sequence summary of MUJV strains 11-1, 11-4, 11-5, 11-16, 11-19 and 11-21 from royal voles (*Myodes regulus*) captured in Korea.

Virus strain	L segment (nt/aa)	M segment (nt/aa)	S segment (nt/aa)
MUJV 11-1	1–6544 (1–2154) JX028271	1–3652 (1–1148) JX028272	1–1831 (1–433) JX028273
MUJV 11-4	1–6544 (1–2154) JX046482	1–3652 (1–1148) JX046483	1–1831 (1–433) JX046484
MUJV 11-5	1~6544 (1–2154) JX046485	1–3652 (1–1148) JX046486	1–1831 (1–433) JX046487
MUJV 11-16	760–4180 (242–1381) JX131287	2045–2805 (670–921) JX131288	1013–1258 (325–405) JX131289
MUJV 11-19	772–4181 (246–1381) JX131290	2045–2809 (670–923) JX131291	1013–1258 (325–405) JX131292
MUJV 11-21	754–4205 (240–1389) JX131293	2045–2805 (670–921) JX131294	1013–1258 (325–405) JX131295

The full-length L-genomic segment of MUJV 11-1, 11-4 and 11-5 was 6544 nucleotides with a predicted RNA-dependent RNA polymerase of 2154 amino acids, starting at nucleotide position 37 and including 43 nucleotides of the 3'-noncoding region (NCR). The entire L-genomic segment of MUJV, compared with HOKV and PUUV, exhibited sequence similarities of 77.7%–79.3% and 90.5%–93.2% at the nucleotide and amino acid levels, respectively (Table 2). The MUJV L genomic segment had six fewer nucleotides and two fewer amino acids than the PUUV L segment [11]. Like other hantaviruses, six major conserved motifs were found in the MUJV L segment: pre-motif, motif A, B, C, D and E [12,13]. A conserved lysine and two arginine residues were observed in pre-motif A. Motif A had a conserved aspartic acid residue, except for asparagine in PUUV Umeå [14]. One glycine and two aspartic acid residues were found in motif B and C, respectively, and glycine and lysine residues were found in motif D. Motif E contained the EFLS site, defined by glutamic acid (E), phenylalanine (F), leucine (L) and serine (S).

The full-length M genomic segment of MUJV 11-1, 11-4 and 11-5 was confirmed to be 3652 nucleotides with a predicted glycoprotein precursor (GPC) of 1148 amino acids. The highly conserved WAASA amino acid motif of the M segment was found at amino acid positions 654–658. Compared with MUJV strain 11-1, the full-length M-segment sequences of PUUV strains differed by 21.6%–23.0% at the nucleotide level and 9.3%–11.1% at the amino acid level (Table 2). At nucleotide positions 62–79, MUJV strains 11-1, 11-4 and 11-5 were longer than MUJV strain 04-4 by 18 nucleotides, which served to distinguish MUJV strains from Inje and Pyeongchang counties in Gangwon province with those from Muju county in Jeollabuk province. At the amino acid level, six amino acids were added at positions 8–13 for MUJV strains 11-1, 11-4 and 11-5.

The entire S segment of MUJV 11-1, 11-4 and 11-5 was 1,831 nucleotides, with a predicted nucleocapsid protein (Np) of 433 amino acids. The sequence similarity among MUJV strains 11-1, 11-4 and 11-5 based on full-length genomes were 98.3%–99.7% and 99.8%–100% at the nucleotide and amino acid levels, respectively. Compared with MUJV 96-1, 99-27, 99-28 and 00-18, 85.7%–86.6% similarity rates at the nucleotide level and 97.9%–99.1% at the amino acid level were found. The NCR was 26 nucleotides shorter than in MUJV 96-1, 99-27, 99-28 and 00-18. The Np amino acid sequence of MUJV strain 11-1 differed from that of HOKV strains by 4.8%–5.1% and PUUV strains by 5.5%–7.2% (Table 2).

**Table 2:** Nucleotide and amino acid sequence similarities (%) of the full-length S, M and L segments of MUJV strain 11-1 and other hantaviruses.

Virus strain	S segment		M segment		L segment	
	1299 nt	433 aa	3444 nt	1148 aa	6462 nt	2154 aa
MUJV 11-16	96.3	97.6	98.3	99.6	98.2	99.6
MUJV 11-19	97.2	98.8	99.2	99.6	98.5	99.8
MUJV 11-21	96.7	98.8	98.3	98.8	98.3	99.7
MUJV 97-32	90.4	98.6	91.7	96.0	-	-
MUJV 04-11	96.6	98.6	-	-	-	-
MUJV 11-4	98.3	99.8	98.3	99.2	98.2	99.8
MUJV 11-5	98.5	99.8	98.2	99.1	98.3	99.9
MUJV 96-1	86.4	98.2	85.9	98.8	-	-
MUJV 96-5	-	-	86.7	98.8	-	-
MUJV 99-7	-	-	86.7	100.0	-	-
MUJV 99-27	86.8	98.8	87.1	98.8	-	-
MUJV 99-28	86.7	98.6	87.1	98.8	-	-
MUJV 00-18	86.8	98.8	86.7	98.8	-	-
MUJV 04-4	82.7	100.0	84.2	97.5	-	-
HOKV Kitahiyama128	82.0	95.2	78.6	92.3	79.3	93.2
HOKV Kamiiso-8Cr-95	81.9	95.2	-	-	-	-
HOKV Tobetsu-60Cr-93	81.9	94.9	-	-	-	-
HOKV Sakhalin99	81.4	95.2	-	-	-	-
PUUV Sotkamo	79.4	94.2	77.6	88.9	78.7	91.6
PUUV Kazan	79.5	93.3	77.3	90.6	78.5	92.4
PUUV Umeå	81.8	94.5	77.0	89.1	77.7	90.5
PUUV Samara49	80.0	93.3	78.3	90.7	78.5	92.3
PUUV Samara94	80.2	93.3	77.8	90.1	78.3	92.2
PUUV CG1820	80.1	92.8	77.8	88.9	78.3	92.0
PUUV Pieksamaki/Mg7	77.3	94.5	78.4	89.7	78.7	91.9
PUUV Pieksamaki/Mg4	-	-	-	-	78.7	91.9
PUUV Pieksamaki/hu-lu	77.3	94.5	78.4	89.7	78.7	91.9
PUUV Pieksamaki/hu-ki	77.3	94.5	78.4	89.7	78.7	91.9
KBRV MF-43	77.4	87.1	74.0	84.0	76.5	90.9
TOPV Ls136V	77.5	87.5	74.6	84.8	77.1	89.1
LUXV LX309	72.2	74.9	68.7	72.2	72.3	79.0
PHV PH-1	74.8	80.1	70.9	76.4	73.4	83.4
TULV 5302v	74.3	79.5	72.9	79.5	75.2	85.8
HTNV 76-118	66.3	62.5	60.5	55.0	67.8	68.5
SEOV 80-39	67.7	62.7	60.8	54.0	67.3	68.2
SOOV SOO-1	66.5	61.3	60.4	54.4	68.0	68.4
DOBV Greece	66.6	61.1	60.1	53.8	68.3	69.4
ANDV Chile9717869	72.0	74.3	65.8	66.3	71.1	76.8
SNV NMH10	71.0	71.3	65.7	67.5	71.2	77.5
MJNV Cl05-11	48.0	47.0	40.7	42.1	65.4	61.9
TPMV VRC66412	49.7	44.9	40.5	42.4	63.9	61.4
NVAV MSB95703	59.0	53.3	56.9	44.1	65.2	61.4

Abbreviations: ANDV, Andes virus; DOBV, Dobrava virus; HOKV, Hokkaido virus; HTNV, Hantaan virus; KBRV, Khabarovsk virus; LUXV, Luxi virus; MJNV, Imjin virus; MUJV, Muju virus; NVAV, Nova virus; PHV, Prospect Hill virus; PUUV, Puumala virus; SEOV, Seoul virus; SNV, Sin Nombre virus; SOOV, Soochong virus; TOPV, Topografov virus; TPMV, Thottapalayam virus; TULV, Tula virus. nt, nucleotides; aa, amino acids.

Between amino acid residues 231 and 325 was the Np hyper-variable region. The Np of MUJV strains 11-1, 11-4 and 11-5 differed by only one amino acid: I to V at position 260. Comparison between MUJV strains from Gangwon and Jeollabuk provinces [8] showed 4 or 5 amino acid differences; amino acid residues AD were replaced with VE or SE at positions 233–234, and an S residue was replaced by N at position 272. Using software available on the @NPS structure server [10], the predicted secondary structure of the MUJV Np was virtually indistinguishable from that of PUUV and HOKV, but was distinctly different from Luxi virus (LUXV) in the Yunnan red-backed vole (*Eothenomys miletus*) (Figure 1B).

**Figure 2:**
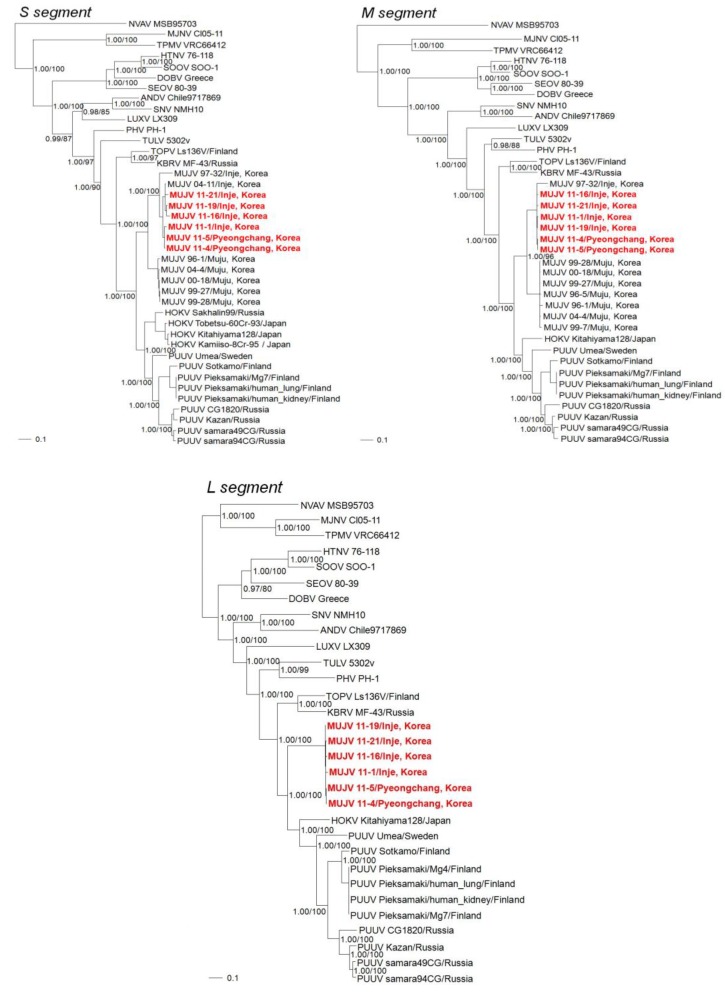
Phylogenetic trees were generated by the maximum-likelihood and Bayesian methods, using the GTR + I + Γ model of evolution, based on the alignment of the S-, M- and L-segment sequences of MUJV strains. Since tree topologies were very similar using RAxML and MrBayes, the trees generated by MrBayes were displayed. The phylogenetic positions of MUJV (96-1: DQ138133, DQ138132; 96-5: DQ138134; 97-32: DQ138136, DQ138125; 99-7: DQ138137; 99-27: DQ138140, DQ138139; 99-28: DQ138142, DQ138141; 00-18: DQ138128, DQ138127; 04-4: DQ138129, EF198313; 04-11: DQ138131) are shown in relationship to arvicolid rodent-borne hantaviruses, including Puumala virus (PUUV Sotkamo: NC_005224, NC_005223, NC_005225; Kazan: Z84204, Z84205, EF405801; Samara49CG: AB433843, AB433850, AB574183; Samara94CG: AB433845, AB433852, AB574184; Umeå: AY526219, AY526218, AY526217; CG1820: M32750, M29979, M63194; Pieksamaki/Mg7: JN831943, JN831944, JN831945; Pieksamaki/Mg4: JN831946; Pieksamaki/human lung: JN831947, JN831948, JN831949; Pieksamaki/human kidney: JN831950, JN831951, JN831952), Hokkaido virus (HOKV Kitahiyama128: AB675463, AB676848, AB712372; Sakhalin99: M5302v: NC_005227, NC_005228, NC_005226), Prospect Hill virus (PHV PH-1: Z49098, AB675453; Tobetsu-60Cr-93: AB010731; Kamiiso-8Cr-95: AB010730), Tula virus (TULV X55129, EF646763), Topografov virus (TOPV Ls136v: AJ011646, AJ011647, AJ011649), Khabarovsk virus (KBRV MF-43: U35255, AJ011648, AJ011650), Luxi virus (LUXV LX309: HM756286, HM756287, HQ404253). Other rodent-borne hantaviruses included Hantaan virus (HTNV 76–118: NC_005218, Y00386, NC_005222), Soochong virus (SOOV SOO-1: AY675349, AY675353, DQ562292), Dobrava virus (DOBV Greece: NC_005233, NC_005234, NC_005235), Seoul virus (SEOV 80-39: NC_005236, NC_005237, NC_005238), Andes virus (ANDV Chile9717869: NC_003466, NC_003467, NC_003468) and Sin Nombre virus (SNV NMH10: NC_005216, NC_005215, NC_005217). Mole and shrew-borne hantaviruses include Nova virus (NVAV MSB95703: FJ539168, HQ840957, FJ593498), Thottapalayam virus (TPMV VRC66412: AY526097, EU001329, EU001330) and Imjin virus (MJNV Cl05-11: EF641804, EF641798, EF641806). The numbers at each node are posterior node probabilities based on 150,000 trees (**left**) and bootstrap values of 1,000 replicates executed on the RAxML BlackBox web server (**right**), respectively. The scale bars indicate nucleotide substitutions per site.

Phylogenetic trees based on full-length L, M and S segments indicated that the MUJV strains in this and a previous study formed a separate lineage, distinct from that of HOKV and PUUV (Figure 2). Moreover, MUJV strains 11-1, 11-4 and 11-5 were clustered with previously analyzed MUJV strains and were genetically distinct from other hantaviruses. Inje and Pyeongchang counties in Gangwon province, the capture sites of MUJV 11-1, 11-4 and 11-5, are located approximately 230 km from Muju county in Jeollabuk province, the capture sites of MUJV 96-1, 99-27, 99-28, 00-18 and 04-4. The MUJV strains showed 13.4%–16.0% nucleotide sequence variation in M and/or S segments. MUJV strains from Gangwon and Jeollabuk provinces could be distinguished phylogenetically (Figure 2). That is, MUJV strains showed geographic-specific clustering based on the capture sites in Gangwon and Jeollabuk provinces (Figure 2). The trees also showed clustering of PUUV strains according to geography. Moreover, MUJV, PUUV and HOKV all shared a common ancestry in trees based on each genomic segment (Figure 2).

## 3. Discussion

According to the ICTV criteria, a hantavirus species is defined as having (I) at least a 7% amino acid sequence difference in the complete Np and GPC; (II) a unique ecological niche of the reservoir host; (III) at least a 4-fold difference in two-way cross-neutralization tests; and (IV) no naturally occurring reassortants [1]. In applying these criteria to MUJV, the following points can be made: (I) the amino acid sequence differences between the Np of MUJV and PUUV (and HOKV) were less than 7%, whereas the amino acid sequence differences between the GPC of MUJV and PUUV (and HOKV) ranged from 7.7%–11.1%; (II) MUJV is harbored by a rodent host species distinct from that of PUUV (and HOKV); (III) because MUJV has not been isolated in cell culture, two-way cross-neutralization tests with PUUV (and HOKV) cannot be performed; and (IV) there was no evidence of naturally occurring reassortment of MUJV.

Because the prevailing analysis does not afford a definitive conclusion, we favor a conservative stance that MUJV might represent a genetic variant of PUUV, rather than being a distinct hantavirus species. The “7%-difference rule”, which was derived at a time when far less was known about the host range and genetic diversity of hantaviruses, may need to be re-examined, particularly in view of findings that the amino acid sequence differences of the Np and GPC between PUUV strains may be up to 8% and 11%, respectively [15], thus exceeding the ICTV criterion of 7%. In this regard, Maes and colleagues proposed that amino acid sequence differences of 10% and 12% or more in the Np and GPC, respectively, should be used to define a distinct hantavirus species [16]. Although the ICTV has not adopted these criteria, we found, in applying these proposed guidelines, that the overall amino acid sequence similarity between the Np and GPC of MUJV, HOKV and PUUV is compatible with MUJV and HOKV being genetic variants of PUUV.

The criterion requiring a unique ecological niche may also need to be reassessed. That is, while MUJV, HOKV and PUUV differ in their rodent hosts, their reservoirs belong to the same family, subfamily and genus. Also, the habitats where these *Myodes* species are found in separate geographic locations are essentially the same, and the rodent species themselves are closely related from the standpoint of their genetics and phylogeny. Moreover, the hantaviruses they harbor are very similar, as judged by their protein sequences. In addition, *My. rufocanus*, the host of HOKV, also serves as the rodent reservoir of PUUV in Scandinavia [17,18]. In fact, some hantavirus species appear to be well established in several rodent species. Notably, Tula virus (TULV) has been found in the common vole (*Microtus arvalis*), Russian common vole (*Microtus* *rossiaemeridionalis*), field vole (*Microtus agrestis*), European pine vole (*Pitymys subterrraneus*) [19,20,21,22,23] and Eurasian water vole (*Arvicola amphibius*) [24]. Also, in South America, some hantavirus species, such as ANDV, Laguna Negra virus and Rio Mamore virus, exhibit a rich diversity of sigmodontine rodent reservoir hosts [25].

Part of the confusion in hantavirus taxonomy lies in the fact that several hantaviruses which have been assigned species status by the ICTV do not fulfill all four criteria. For example, El Moro Canyon virus, Isla Vista virus, Muleshoe virus and Rio Segundo virus are classified as hantavirus species by the ICTV, but none has been isolated in cell culture. As such, as with MUJV, two-way cross-neutralization tests cannot be performed. In addition, Muleshoe virus has been detected in the hispid cotton rat (*Sigmodon hispidus*), but so has Black Creek Canal virus, yet both viruses, despite their Np sequences differing by less than 7%, are listed as separate hantavirus species. Moreover, Topografov virus (TOPV), isolated from the Siberian lemming (*Lemmus sibiricus*) [26], is the same virus as Khabarovsk virus (KBRV), originally detected in the reed vole (*Microtus fortis*) and now believed to be hosted by the Maximowiczii’s vole (*Microtus maximowiczii*) [27,28], but both TOPV and KBRV are classified as separate hantavirus species by the ICTV.

Another confusing example is Saaremaa virus [29], which does not meet the 7% amino acid difference in the Np and GPC criterion or the unique ecological niche requirement. That is, the Np and GPC amino acid sequences of Saaremaa virus differ from that of DOBV by only 3%, and Saaremaa virus is hosted by the striped field mouse, which is the same rodent reservoir species of HTNV in Asia, again calling into question the notion that each hantavirus species occupies a unique ecological niche. Recently, in a detailed study of DOBV, a consortium of European hantavirus experts concluded that Saaremaa virus was not a distinct hantavirus species. Instead, four genotypes of DOBV were proposed: namely Dobrava, Saaremaa, Kurkino and Sochi [30]. A subsequent independent study also arrived at the same conclusion [31].

Although HFRS cases have not been definitively associated with MUJV, approximately 5% of HFRS patients in Korea exhibit significantly higher antibody titers against PUUV than to HTNV or SEOV, suggesting that a PUUV-like hantavirus, such as MUJV, carried by a *Myodes* species, may be pathogenic in Korea [8]. In the absence of a MUJV isolate, confirmation by plaque-reduction neutralization testing is not currently possible. However, it may be premature to conclude that MUJV represents a nonpathogenic hantavirus. Also, not all strains of a given hantavirus species show the same degree of pathogenicity. For example, the Saaremaa genotype of DOBV is distinctly less pathogenic than the Dobrava and Sochi genotypes [30]. Accordingly, MUJV, and possibly HOKV, may represent attenuated or less pathogenic genotypes of PUUV.

## 4. Experimental

Royal voles were captured using Sherman traps (Sherman, H.B., Tallahassee, FL, USA) at six sites in two central provinces in Korea during 2008–2011 (Figure 1A). Lung and spleen tissues of royal voles, dissected using sterile instruments, were frozen at −70 °C until use for gene amplification and virus isolation.

Sera from royal voles were examined for IgG antibodies against PUUV by the indirect immunofluorescent antibody (IFA) test. Briefly, sera were incubated with PUUV-infected Vero E6 cells spotted on microscope slides, and virus-specific fluorescence was detected using a fluorescent microscope (Axioscope, Zeiss, Berlin, Germany).

For virus isolation, 10% (*w*/*v*) homogenates of lung and/or spleen from royal voles, confirmed as hantavirus infected by IFA and RT-PCR, were inoculated onto Vero E6 cells (ATCC C1008 CRL-1586). Also, suckling hamsters were injected with tissue homogenates by the intracerebral route.

For genetic analyses of MUJV, total RNA was extracted from 50–100 mg of lung tissue using RNA-Bee solution (Tel-Test Inc., Friendswood, TX, USA), and oligonucleotide primers were designed using the MegAlign Clustal W program (DNASTAR Inc., Madison, WI, USA). Newly designed primers with mixed bases were used for conventional PCR and DNA sequencing. Generally, the primer length was 18–22 base pairs for adequate specificity and complementary binding to the DNA template. The GC content (or the percentage of G and C bases) in each primer was 40%–60%.

cDNA was synthesized using M-MLV reverse-transcriptase (Promega, Madison, WI, USA) with a highly conserved primer and/or random hexamers by two-step RT-PCR cycles. The 5'- and 3'-termini of segments were amplified using the 3'-Full RACE Core Set (Takara Bio Inc., Otsu, Japan). For taxonomic identification, mtDNA was extracted from the liver of royal voles, using the High Pure Template Preparation Kit (Roche, Indianapolis, IN, USA). Nucleotide and amino acid sequences of each segment of MUJV were compared with PUUV [11,14,32], HOKV [6,33], LUXV [34], Prospect Hill virus [35], KBRV [27], TULV [36], SNV [37], HTNV [38], DOBV [39], Soochong virus [40], SEOV [41], and previously sequenced MUJV strains [8].

Phylogenetic trees were generated by maximum likelihood and Bayesian methods, implemented in PAUP* (Phylogenetic Analysis Using Parsimony, 4.0b10) [42], RAxML Blackbox webserver [43] and MrBayes 3.1 [44], under the best-fit GTR + I + Γ model of evolution selected by hierarchical likelihood-ratio test in MrModeltest v2.3 [45] and jModelTest version 0.1 [46]. Two replicate Bayesian Metropolis-Hastings Markov Chain Monte Carlo runs, each consisting of six chains of 10 million generations sampled every 100 generations with a burn-in of 25,000 (25%), resulted in 150,000 trees overall. The complete S, M and L segments were treated separately in phylogenetic analyses. Topologies were evaluated by bootstrap analysis of 1000 iterations, and posterior node probabilities were based on 2 million generations and estimated sample sizes over 100 (implemented in MrBayes [44]).

## 5. Conclusions

As determined by double-sandwich IgM-capture enzyme immunoassay, approximately 5% of HFRS cases in Korea exhibit four-fold or higher antibody titers to PUUV than to HTNV [8], suggesting that a PUUV-related hantavirus, such as MUJV, may be pathogenic. Unfortunately, all attempts to isolate MUJV in Vero E6 cells and suckling hamsters have been unsuccessful. Recently, MRK101, a newly established cell line derived from kidney tissue of the grey red-backed vole, was used to propagate HOKV, ending a nearly two-decade effort [33]. Investigations are now being planned to employ this cell line, as well as to establish cell lines from royal vole kidney tissues, to isolate MUJV. The availability of a MUJV isolate will make possible specific serodiagnosis of HFRS cases in Korea that are suggestive of PUUV-related hantavirus infection. Also, a MUJV isolate would facilitate studies aimed at further clarifying its phylogeography, as well as the evolutionary relationships among hantaviruses possibly harbored by other closely related arvicolid rodent species, such as the Shanxi red-backed vole (*My. shanseius*) in China and the Smith's red-backed vole (*My. smithii*) in Japan.

Apart from the need for more sensitive methods to isolate hantaviruses, findings from this study, as well as recent analyses of the rapidly expanding hantavirus sequence database [2], highlight the need to reassess the current ICTV criteria for the classification of hantaviruses, particularly those which have yet to be isolated in cell culture. This will become more urgent, as many more hantaviruses are found in shrews, moles and bats, as well as in other unanticipated taxonomic orders of small mammals from widely separated geographic areas.
